# Sleep Disturbance and Rotator Cuff Tears: A Systematic Review

**DOI:** 10.3390/medicina55080453

**Published:** 2019-08-08

**Authors:** Umile Giuseppe Longo, Gabriella Facchinetti, Anna Marchetti, Vincenzo Candela, Laura Risi Ambrogioni, Aurora Faldetta, Maria Grazia De Marinis, Vincenzo Denaro

**Affiliations:** 1 Department of Orthopaedic and Trauma Surgery, Campus Bio-Medico University, 00128 Rome, Italy; 2 Research Unit of Nursing Science, Campus Bio-Medico University, 00128 Rome, Italy

**Keywords:** rotator cuff, rotator cuff tear, sleep quality, sleep disturbance, sleep factor, sleep wake disorders, sleep, shoulder, pain

## Abstract

*Background and Objectives*: Sleep disorders are one of the most common complaints of patients with rotator cuff (RC) tears. However, potential correlations between the treatment of RC tears and the causal factors of sleep disorders are still under discussion. The aim of this review is to evaluate quality of sleep in patients before and after surgery for RC tears and to identify which factors affected patients’ sleep. *Materials and Methods*: A systematic review was conducted. To provide high quality of the review, the included studies were evaluated with the standardized tool “Quality Assessment Tool for Quantitative Studies” developed by the Effective Public Health Practice Project. *Results*: The search strategy yielded 78 articles. After duplicate removal and titles, abstracts and full-texts review, four studies were included in the systematic review. Concerning shoulder function, the most frequently reported scale was the Simple Shoulder Test (SST). Regarding sleep quality, the most frequently reported score was the Pittsburgh Sleep Quality Index (PSQI). *Conclusion*: We found that the majority of patients with RC tears had a sleep disturbance, especially before surgery with a general improvement in sleep quality post-operatively. Moreover, sleep quality was correlated with pain and it also seems that factors as comorbidities, obligatory position during night time, preoperative and prolonged postoperative use of narcotics and psychiatric issues may play an important role in sleep quality.

## 1. Introduction

Epidemiological studies have shown that sleep disorders involve 15% to 35% of the general population [1,2]. Sleep disorder is a medical disorder of the sleep patterns that may be serious enough to interfere with normal physical, mental, social, and emotional functioning and it negatively affects patient’s quality of life, causing mood disorders and anxiety [3,4]. Sleep a few hours at night can have consequences on emotional condition [3,5] and on the ability to perform daily life activities [6].

Rotator cuff (RC) tears are frequent [7,8] and they have a multifactorial etiology [8,9,10,11,12]. Patients with symptomatic RC tears can experience negative impacts upon all areas of their lives [6], complaining of functional limitations and reporting depression, anxiety, and sleep disturbance [13].

Sleep disturbance is one of the most common complains of patients with RC disease [1,3], with only 11% of patients with symptomatic RC tears claiming to have a normal sleep [1,14]. Patients with RC tears seems to have more sleep disturbances compared with patients with other shoulder disorders like RC tendinopathies [3], or subacromial impingement [4].

Shoulder pain increases at night typically. For this reason, the quality of sleeping in these patients is scanty, even though the degree of pain preventing patients sleeping is unknown [4,15]. An increased production of inflammatory cytokines has been indicated as a possible causative factor [4]. Even though RC repair improves psychological status, physical function, and general health [13], patients may still complain of sleep disturbance. Different causes that can play an important role in decreasing sleep quality include functional deficits, maintaining a forced position, and shoulder stiffness [1].

In fact, two years after arthroscopic RC repair, 41% of patients report having sleep disorders [14]. Furthermore, it has been observed that some patients who have sleep disorders after RC surgery can benefit from conservative treatment such as physiotherapy or medication [3,6].

Despite the increasingly evident association between sleep disorders and RC tears, few studies investigate a possible link between the treatment of RC tears and the potential causal factors of sleep disorders. To date it has been shown that there is a relationship between the quality of sleep in patients with RC tears and factors such as hypertension, depression, female sex, low back pain, diabetes mellitus, body mass index (BMI), previous corticosteroid injection, and osteoarthritis [3]. However, no statistically significant correlations were found between any of the features of the RC tear and sleep quality [5].

Therefore, the aim of this review is to evaluate the quality of sleep in patients before and after surgery for RC tears and to identify which factors affected patients’ sleep.

## 2. Materials and Methods

A systematic review was conducted using the Preferred Reported Items for Systematic Review and Meta-analysis Statement (PRISMA) guidelines [16]. Although systematic review is a traditional method to assess the effectiveness of treatment, systematic reviews can also provide evidence about prevalence and/or incidence, diagnostic test accuracy, etiology and/or risk, and prognosis [17]. To identify relevant studies, medical subject headings and free-terms were searched for the following keywords “rotator cuff”, “rotator cuff tear”, “sleep quality”, “sleep disturbance”, “sleep factor”, “sleep wake disorders”, “sleep”, “shoulder”, “pain”. Data were collected from January 2019 to May 2019 using the following databases: US National Library of Medline (PubMed), Cumulative Index to Nursing and Allied Health Literature (CINAHL), Cochrane, and Embase. The inclusion criteria were (1) sleep disturbance or sleep disorders in patients with RC tears before and after surgery measured with at least one score; (2) any potential physical, psychological, and emotional risk factors for sleep disturbance; (3) evaluation of sleep disturbance with validate instrument for sleep. Missing data pertinent to these parameters warranted exclusion from this systematic review. Three independent reviewers separately conducted the search (AF, GF, UGL). All peer-reviewed journals were considered, and all relevant studies were analyzed. Based on the inclusion criteria study, all articles retrieved were independently screened for abstract and full-text by three authors. Any differences were resolved by discussion until consensus was achieved. A cross-reference search of the selected articles was also performed to obtain other relevant articles for the study. In addition, the reference lists of included articles were manually examined to identify potentially relevant studies that were not retrieved with the initial search. Level I-IV articles in accord with the Oxford Centre of Evidence Based Medicine (EBM) were found in the literature and included in the study. Literature reviews, case reports, studies in vitro, technical notes, letters to editors, and instructional course materials were also excluded. Finally, to avoid bias, the selected articles, the list of references, and the articles excluded from the study were reviewed, assessed, and discussed by all the authors. If there was disagreement among authors regarding the inclusion and exclusion criteria, the senior investigator made the final decision. No meta-analysis was performed given the insufficient data available from the literature.

### Risk of Bias Assessment

To provide a high quality review, the included studies were evaluated with the standardized tool “Quality Assessment Tool for Quantitative Studies” developed by the Effective Public Health Practice Project (EPHPP) (Table 1) [18]. The tool provides a standardized means to assess study quality with an overall methodological rating of strong, moderate or weak in eight sections: selection bias, study design, confounders, blinding, data collection methods, withdrawals and dropouts, intervention integrity, and analysis. Moreover, the tool allows to develop recommendations for study findings.

## 3. Results

**Figure 1:**
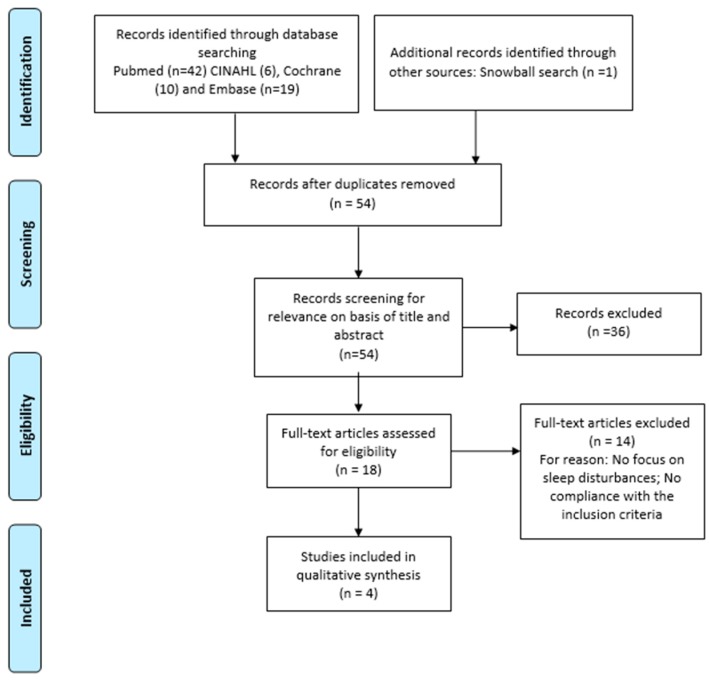
Preferred Reported Items for Systematic Review and Meta-analysis Statement (PRISMA) 2009 flow diagram.

The selection process is illustrated in Figure 1. The search strategy yielded 78 articles. After duplicate removal and titles, abstracts and full-texts review, four studies [1,2,14,19] were included in this systematic review.

### 3.1. Study Characteristics

The four studies included were published from 2015 to 2018 and were conducted in the USA (*n* = 2) [1,14], Turkey (*n* = 1) [2], and Korea (*n* = 1) [19]. All articles were published in English in peer-reviewed journals. All participants in the studies had RC tears and had undergone a surgical and no surgical approach.

### 3.2. Demographics

A total of 171 shoulders in 171 patients were reported of which 56 were males and 78 females, with a mean age of 59.3 (SD 21.9, range 18–78) (Table 2). The arm involved in the trauma was specified in only two of four studies [2,19]. Patients were assessed at an average follow-up period of 6 months ranging from 2 weeks [1,14] to 24 months [14]. A surgical procedure was performed in all of the studies.

### 3.3. Outcome Measurements

Several scales were reported in the included studies. Details are reported in Table 3 and Table 4.

### 3.4. Shoulder Function

The most frequently reported scale was the Simple Shoulder Test (SST) (*n* = 2) [1,14]. Other scales used were the University of California Los Angeles Scale (UCLA) (*n* = 1) [19], the Western Ontario Rotator Cuff Scale (WORC) (*n* = 1) [2], and the Constant and Murley Shoulder Score (CSS) (*n* = 1) [2]. These scores were administered to patients before and after treatment.

### 3.5. Sleep Quality

The most frequently reported score was the Pittsburgh Sleep Quality Index (PSQI) that was used in all four articles [1,2,14,19]. This index is a standardized self-reported questionnaire consisting of 19 items summarized in subjective quality of sleep, sleep latency, sleep duration, habitual sleep efficiency, sleep disturbances, use of sleep medications, and daytime dysfunction. A score from 0 (no sleep problem) to 3 (most frequent sleep problem) is assigned. The overall PSQI score is obtained from the sum of the components, ranging from 0 to 21. PSQI was greater than 5 in all of the articles before treatment (indicative of poor sleep). Moreover, it decreased after surgical or conservative treatment in all four studies [1,2,14,19]. The questionnaire was also administered to patients after treatment with 24 months follow-up period [14].

### 3.6. Other Scales

Before and after any interventions, other scales, in addition to the above, were administered to patients such as the World Health Organization Quality-Of-Life Scale Abbreviated Version (WHOQOL-BREF) (*n* = 1) [19], the Hospital Anxiety and Depression Scale (HADS) (*n* = 1) [19], and the Visual Analog Scale for Pain (VAS) (*n* = 3) [1,14,19].

## 4. Discussion

Even though sleep disorders are frequently associated with impairment in patients with RC tears, the correlation between sleep disturbance and RC tears is not well documented, and causative factors are still unknown. In this systematic review of four trials, we found that the majority of patients with RC tears had a sleep disturbance [1,2,14,19] and they completed a PSQI score. An increased PSQI (PSQI > 5 indicative of poor sleep) before surgery was observed, with a general improvement in sleep quality post-operatively [1,2,14,19], even though the nature of improved sleep remained unclear. Sleep quality was correlated with pain, as evaluated using the VAS: an increased VAS score seems to be associated with an increased PSQI score [1,14,20]. However, the possibility of pain decreasing because sleep is improving requires further studies. Sleep disturbance is multifactorial [1], and several factors may influence sleep quality in patients with RC tears: lower back pain, diabetes mellitus, sling use, shoulder stiffness, an obligatory position during night time [1], and preoperative and prolonged postoperative use of narcotics [1,14].

Moreover, the use of morphine increases wakefulness [1,21], and psychiatric issues may also be related to sleep disturbance [5], but it remains unclear whether these represent a causal factor. Poor sleep quality in patients with RC disease was correlated with the presence of depression, which was not present in other shoulder pathologies [4,5,13]. This suggests that in these categories of patients, sleep disturbance may result from other sources that are yet to be identified [3]. RC repair may improve sleep quality, for instance, after surgery, negative emotional states such as depression and anxiety decreased [19]. Moreover, RC surgery enabled a substantial reduction in pain and an increase in functional capacity [2]. Data from available articles were not univocal. For example, patients started sleeping better 3 months after undergoing arthroscopic rotator repair [1,14], continued to report normal sleep quality after 6 months [1,14], and around 40 [1,14] patients continued to report sleep disturbance after RC surgery, which is similar to the rate reported in the general public (35%). Contradictory results have been found in efforts to correlate nocturnal pain with specific shoulder disorders. In a cross-sectional epidemiological study, 71% of patients with RC tears, 85% of patients with osteoarthritis and 86% of patients with adhesive capsulitis reported a PSQI score of >5 [4]. However, Cho et al. found no significant differences between patients with shoulder pathologies (including those with RC tears, adhesive capsulitis, and calcific tendinitis) in terms of mean scores on the PSQI and VAS [22]. The factors explaining the increased levels of nocturnal pain and the higher PSQI scores in patients with RC tears are still debated [23]. Synovial inflammation (in the glenohumeral joint and subacromial space) and increased levels of proinflammatory and pain-related cytokines are common features of shoulder disorders, including RC tears [4,24]. Peak melatonin levels during the night and early morning may activate this inflammatory response [23] and may be responsible for the increase in nocturnal pain levels [4]. There was no correlation between the level of pain and the characteristics of the RC lesion (except medial retraction) based on magnetic resonance imaging [5,25]. The only factors that might have been related to pain were increased comorbidities, education level, race, poor mental health, and advanced age [25,26]. The size of the lesion did not correlate with sleep disturbance [1,3]. Sleeping position may correlate with sleep quality in patients with RC disease, as the sleeping position influences subacromial pressure [27]. A supine position causes significantly lower subacromial pressure than being in a prone or lateral decubitus position, and it leads to the propagation of degenerative changes, atrophy, and tear progression [27]. A full-thickness RC tear may have a protective effect in terms of dissipating pressure between the subacromial space and the glenohumeral joint space [3]. Ninety-six percent of substance abusers reported sleep disturbance [2]. The preoperative use of narcotic pain medication and the prolonged postoperative use of narcotics are known to disrupt the normal sleep cycle, resulting in decreased rapid eye movement sleep and increased wakefulness [14]. PSQI scores in patients using narcotic pain medication was higher at 6 months postoperatively [1], increasing dramatically at two years postoperatively, with an increased PSQI score of 7.4 points in narcotic user patients than those not using narcotics [14]. Moreover, in a study which evaluates sleep and postoperative pain, it was shown that with drug treatment the pain was adequately controlled in all patients and, furthermore, that no significant influence on the objective measurement of postoperative sleep was found. In addition, despite the absence of opioids or significant pain, postoperative patients experienced a profound sleep disorder. Furthermore, morphine use increased wakefulness and inhibited rapid eye movement (REM) sleep [21]. This finding suggests that counselling patients to discontinue narcotic use preoperatively and to limit postoperative consumption may decrease sleep disorders after RC surgery [1]. Surgical factors, such as the number of anchors needed to repair the RC, or the addition of other procedures (tenodesis or tenotomy of the biceps, acromioplasty, or distal clavicle resection) did not affect sleep disturbance [1].

This systematic review has some limitations. First, the studies utilized different scores to evaluate the shoulder function, such as the SST [1,14], the ASES score (*n* = 1) [19], and the VAS score (*n* = 3) [1,14,19]. All studies utilized the PSQI score for the evaluation of sleep quality [1,2,14,19]. All studies included in this review reported different scales for evaluating sleep disorders making it difficult to compare them. Moreover, the measurement tools reported subjective patient measures, making it difficult to obtain an objective analysis of sleep habits [2,4,14]. Second, a relatively small sample of patients with RC tears were analyzed, and in each study sample. Third, the time points of the included studies varied. The results of some included studies were based on outcome questionnaires collected at a single time point before any clinical treatment; therefore, it was impossible to draw conclusions regarding how treatment may have influenced these findings [3,4,5,6]. Fourth, few studies included information on the etiopathogenetic factors responsible for the sleep disturbance in these patients. The majority of the included studies were level-III evidence studies or lower, relegating the review to the inherent limitations of this level of evidence. Selection bias was evident in the different patient populations, based on several continuous and categorical variables: gender, age, and arm dominance. Fifth, there is a lack of imaging to document the healing of the RC, therefore making it impossible to understand whether improvement in sleep correlated with biological healing of the RC [1,2,4,6,14,19,28]. Finally, the studies included in this review do not report potential factors that can affect the patient’s sleep and, therefore, we are unable to compare our results with those of the studies.

## 5. Conclusions

The majority of patients with RC tears had a sleep disturbance, especially before surgery with a general improvement in sleep quality post-operatively. Moreover, sleep quality was correlated with pain as risk factors, and it also seems that factors as comorbidities, obligatory position during night time, preoperative and prolonged postoperative use of narcotics, and psychiatric issues may play an important role in sleep quality. Furthermore, this review also highlighted the profound lack of studies in this area and, therefore, further studies are needed to assess the possible risk factors of sleep quality and sleep disturbances in patients with RC tears.

## Figures and Tables

**Table 1:** Effective Public Health Practice Project (EPHPP)—Quality Assessment.

Authors, Year	Global Rating	Selection Bias	Study Design	Confounders	Blinding	Data Collection Methods	Withdrawals and Drop-Outs
Austin et al. 2015	Weak	Strong	Weak	Weak	Weak	Strong	Strong
Cho et al. 2015	Weak	Weak	Weak	Strong	Moderate	Strong	Strong
Horneff et al. 2017	Weak	Weak	Weak	Weak	Weak	Strong	Moderate
Serbest et al. 2017	Weak	Weak	Weak	Strong	Moderate	Strong	Strong

**Table 2:** Characteristic of included studies.

Author	Country	Study Design (Level of Evidence)	Type of Treatment	Patient (N)	Shoulder	Mean Age (y) (Range)	Trauma Side (D/ND; R/L/B)	Gender (Male/Female)	Time of Follow Up (Months)
Austin et al. 2015 [1]	USA	Case series (IV)	Arthroscopic rotator cuff repair	56	56	59.8 (45–78)	-	27/29	0.5–1.5–3–4.5–6
Cho et al. 2015 [19]	KOREA	Retrospective study (II)	Surgical treatment	47	47	57 (43–75)	D32/ND15	20/27	3–6–12
Horneff et al. 2017 [14]	USA	Case series (IV)	Arthroscopic rotator cuff repair	37	37	>18 years	-	-	0.5–1.5–3–4.5–6–24
Serbest et al. 2017 [2]	TURKEY	Retrospective study (II)	Surgical treatment	31	31	61 (26–75)	R18/L13	9/22	6

- = missing data; N = number; Y = year; D/ND = dominant/not dominant; R/L/B = right/left/both.

**Table 3:** Scales used and follow-up scores.

	Scales	Follow-Up (mo)	Austin et al. 2015 [1]	Cho et al. 2015 [19]	Horneff et al. 2017 [14]	Serbest et al. 2017 [2]
**OULDER FUNCTION**	**ASES**	Pre-intervention		42.5 ± 16.5		
		0.5				
		1.5				
		3		60.3 ± 16.3		
		4.5				
		6		72.5 ± 13.5		
		12		87.2 ± 10.9		
		>12				
	**UCLA**	Pre-intervention		12.8 ± 4.9		
		0.5				
		1.5				
		3		24.3 ± 4.7		
		4.5				
		6		27.9 ± 3.4		
		12		31.0 ± 3.3		
		>12				
	**SST**	Pre-intervention	4.2		4.3	
		0.5				
		1.5	3			
		3	6			
		4.5	8.8			
		6			9.5	
		12	9.5		10	
		>12			10.8	
	**WORC**	Pre-intervention				80
		0.5				
		1.5				
		3				
		4.5				
		6				36
		12				
		>12				
	**CSS**	Pre-intervention				46
		0.5				
		1.5				
		3				
		4.5				
		6				78
		12				
		>12				
**SLEEP QUALITY**	**PSQI**	Pre-intervention	11.70 ± 4.61	6.6 ± 3.6	11.6 ± 4.4	15
		0.5				
		1.5	11		11.3	
		3	8	5.6 ± 3.9	8.3	
		4.5	6		6.1	
		6		5.8 ± 3.2	6.2	6
		12	6	4.2 ± 3.3		
		>12			5.5	
		0.5				
		1.5				
		3				
		4.5				
		6				
		12				
		>12				
**OTHER SCALE**	**VAS**	Pre-intervention	6	6.7 ± 1.6	5.4	
		0.5				
		1.5	3			
		3	2.8	4.3 ± 2.0		
		4.5	1.8			
		6		2.9 ± 2.1	1.9	
		12	1.9	1.3 ± 1.4	1.7	
		>12			1.2	
	**HADS**	Pre-intervention		D: 3.7 ± 3.3; A: 4.3 ± 4.3		
		0.5				
		1.5				
		3		D: 2.4 ± 3.2; A: 2.5 ± 4.2		
		4.5				
		6		D: 2.4 ± 2.5; A: 2.1 ± 2.9		
		12		D: 2.1 ± 2.3; A: 1.4 ± 2.4		
		> 12				
	**Other**	Pre-intervention		WHOQOL-BREF: 60.4 ± 11.0		
		0.5				
		1.5				
		3		62.4 ± 12.3		
		4.5				
		6		63.6 ± 12.9		
		12		67.4 ± 11.8		
		>12				

ASES: the American Shoulder and Elbow Surgeons score; UCLA: the University of California Los Angeles Scale; SST: Simple Shoulder Test; CSS: the Constant and Murley shoulder score; WORC: the Western Ontario Rotator Cuff Scale; PSQI: the Pittsburgh Sleep Quality Index; VAS: the Visual Analog Scale for Pain; WHOQOL-BREF: the World Health Organization Quality-Of-Life Scale Abbreviated Version; HADS: the Hospital Anxiety and Depression Scale.

**Table 4:** Combination of scales used.

	Shoulder Function					Other *			
	ASES	UCLA	SST	CSS	WORC	PSQI	VAS	WHOQOL-BREF	HADS
Austin et al. 2015 [1]			X			X	X		
Cho et al. 2015 [19]	X	X				X	X	X	X
Horneff et al. 2017 [14]			X			X	X		
Serbest et al. 2017 [2]				X	X	X			

ASES: the American Shoulder and Elbow Surgeons score; UCLA: the University of California Los Angeles Scale; SST: Simple Shoulder Test; CSS: the Constant and Murley shoulder score; WORC: the Western Ontario Rotator Cuff Scale; PSQI: the Pittsburgh Sleep Quality Index; VAS: the Visual Analog Scale for Pain; WHOQOL-BREF: the World Health Organization Quality-Of-Life Scale Abbreviated Version; HADS: the Hospital Anxiety and Depression Scale. * Other scales used but not specific of shoulder function and quality of sleep.
